# Dynamic regulation of RNA editing of ion channels and receptors in the mammalian nervous system

**DOI:** 10.1186/1756-6606-2-13

**Published:** 2009-05-29

**Authors:** Bao Zhen Tan, Hua Huang, Runyi Lam, Tuck Wah Soong

**Affiliations:** 1 Department of Physiology, Yong Loo Lin School Medicine, National University of Singapore, 117597, Singapore; 2 Department of Biomedical Engineering, Johns Hopkins University School of Medicine, Baltimore, MD 21205, USA; 3 National Neuroscience Institute, Jalan Tan Tock Seng, Singapore

## Abstract

The post-transcriptional modification of mammalian transcripts in the central nervous system by adenosine-to-inosine RNA editing is an important mechanism for the generation of molecular diversity, and serves to regulate protein function through recoding of genomic information. As the molecular players and an increasing number of edited targets are identified and characterized, adenosine-to-inosine modification serves as an exquisite mechanism for customizing channel function within diverse biological niches. Here, we review the mechanisms that could regulate adenosine-to-inosine RNA editing and the impact of dysregulation in clinical conditions.

## Introduction

Nuclear pre-mRNA editing by selective adenosine deamination is catalyzed by a family of enzymes known as Adenosine Deaminases Acting on RNA (ADARs) [1], which results in a single nucleotide change from adenosine (A) to inosine (I). A-to-I editing is a dynamic and versatile post-transcriptional mechanism of single nucleotide recoding, which could drastically alter both the functional properties and expression levels of protein-coding mRNAs, increasing the repertoire of proteins available. Currently, most of the identified targets of A-to-I RNA editing are found in the mammalian nervous system, such as the ion channels and neurotransmitter receptors that play crucial roles in electrical excitability and signal transduction. Recoding of these proteins by RNA editing provides an attractive mechanism for customizing specific channel function within diverse biological niches, and any dysregulation of the process may contribute to the pathogenesis of certain diseases. Despite the tremendous amount of research invested in understanding the effects of RNA editing, how this activity is dynamically regulated remains unclear. In this review, we would discuss some potential mechanisms and factors that could dynamically regulate this A-to-I modification by ADAR, namely the expression and activity of ADAR family members via self-editing, alternative splicing and cross-talk between these two mechanisms. Environmental cues and other factors that modulate developmental and activity-dependent RNA editing will also be examined. In addition, some consequences of dysregulation in RNA editing in the nervous system will be discussed with regards to their clinical relevance.

### RNA editing of ion channels and receptors alters their function and expression

Ion channels and neurotransmitter receptors in the mammalian nervous system are more common among the known targets of A-to-I RNA editing by ADARs [1]. Some notable examples include a number of glutamate-gated ion channels, the voltage-gated K_v_1.1 potassium channel, the GABA_A _receptor and the serotonin 5-HT_2C _receptor. In almost all cases, A-to-I editing occurs at precise and functionally important locations in the protein, changing key amino acid residues crucial for the protein function [2]. Table S1 [see Additional file 1] summarizes the recoding of ADAR substrates by RNA editing, the amino acid position, and the physiological changes in channel properties and expression.

### Glutamate-gated ion channels

A-to-I RNA editing of the glutamate-gated ion channels is the most extensively studied [3]. Five subunits of the glutamate receptor (GluR-B, GluR-C, GluR-D, GluR-5 and GluR-6) are found to undergo ADAR-mediated RNA editing [1]. Currently, a total of 4 editing sites that result in amino acid changes have been identified, namely glutamate to arginine (Q/R), arginine to glycine (R/G), isoleucine to valine (I/V), and tyrosine to cysteine (Y/C).

The first target of A-to-I RNA editing discovered in the mammalian system was the AMPA GluR-B subunit mRNA, in which a genomically encoded glutamine codon (CAG) was changed to an arginine codon (CIG) at position 607 [4]. This edited Q/R site, also found in the kainate receptor subunits GluR-5 and -6 [4], resides in the "pore loop region" of membrane segment 2 which determines the ion permeability of the glutamate channel [5]. Channels that contain the edited R form are less permeable to calcium [6]. However, for GluR-6, multiple editing at the Q/R, I/V and Y/C sites, located at positions 621, 567 and 571 respectively, may lead to higher calcium permeability [7]. Editing of the glutamate-gated ion channels at the R/G site changes an arginine (AGA) residue to glycine (IGA), resulting in an enhanced rate of recovery from receptor desensitization [8].

In addition to regulating the electrophysiology of the channel, editing also plays a role in other aspects of channel functions, namely channel assembly, exit from the endoplasmic reticulum and channel inhibition by endogenous substances. The edited GluR-B^R ^isoforms mainly exist as monomers in the endoplasmic reticulum (ER), whereas unedited GluR-B^Q ^isoforms have a higher propensity to tetramerize and be transported to the synaptic membrane [9,10]. Thus, editing at the Q/R site will affect the availability of functional channels at synapse. Editing of the Q/R site in the GluR-5 and GluR-6 subunits affects inhibition of the channel by membrane fatty acids such as arachidonic acid and docosahexaenoic acid [11]. Kainate receptors with only the edited isoforms are strongly inhibited by these compounds, while inclusion of a single unedited copy is sufficient to confer resistance to this inhibition.

In most cases, editing is not 100% efficient and both the edited and non-edited isoforms co-exist in the cell. An interesting exception is editing of the Q/R site at the GluR-B subunits. In transgenic mice, failure to edit at this site leads to epileptic seizure and death within 3 weeks after birth [12].

### Voltage-gated Potassium channel

Bhalla et al [13] reported that the mRNA transcript of human K_v_1.1 undergoes A-to-I RNA editing, resulting in an amino acid change from genomic isoleucine to valine (GTT) [14]. As the hK_v_1.1 gene is intronless, its exon-to-exon pairing forms the RNA duplex that is recognized by ADAR2. The A-to-I RNA editing occurs specifically at position 400 in the channel's sixth transmembrane segment (S6), which lines the ion conducting pore. Electrophysiology analysis shows that the edited hK_v_1.1 exhibits rapid recovery from fast inactivation as compared to the wild type channel.

Interaction of an inactivating particle with the pore of hK_v_1.1 channel results in channel inactivation. The I-to-V amino acid change may disrupt this interaction, resulting in a faster recovery from inactivation [15]. Voltage-gated potassium channel plays an important role in the repolarization phase of the action potential. Therefore, changes in the inactivation kinetics will have an impact on the duration and frequency of the action potential. The faster recovery from inactivation shortens the duration of each action potential and increases the frequency of action potential [15]. To add to the complexity, potassium channels exist as tetramers, and each channel may contain different ratios of edited and unedited subunits. The physiological significance of the different extent of editing on the final properties of channel inactivation and the firing pattern of a neuron has yet to be determined.

### GABA_A _receptor

The isoleucine codon (ATA) of the α3 subunit of the GABA_A _receptor is highly edited, resulting in a methionine codon (ATI) in the adult rat, mouse and human brains [16,17]. This I/M site resides in position 342 in the predicted third transmembrane domain of the α3 subunit. Functional analyses revealed that editing of this mRNA transcript results in GABA_A _receptors with smaller amplitudes, slower activation, and faster deactivation compared to the unedited receptor [17].

GABA_A _receptors are ligand-gated chloride channels, consisting of 5 subunits – 2 α subunits, 2 β subunits, and either a γ or δ subunit [18]. In the adult brain, activation of the GABA_A _receptors causes an influx of chloride ions, which produces an inhibitory current. However, during development, activation of the GABA_A _receptors produces an excitatory response, which is important for development of the nervous system circuitry [19]. Editing of the α3 subunit is developmentally regulated – the edited GABA_A _receptors are expressed predominantly in the mature adult brain while the unedited receptors are mostly found in the developing brain [17]. Hence, RNA editing may regulate the function of the GABA_A _receptors during development – by allowing the expression of the non-edited GABA_A _receptors at the early stages, which play a crucial role in synaptic formation, followed by expression of the edited subunit in adulthood that is more suited to the inhibitory functions of GABA_A _receptors in action potentials.

### Serotonin 2C receptor

The 2C subtype of serotonin receptor (5-HT_2C_R), a member of the G protein-coupled receptor superfamily, undergoes A-to-I RNA editing at five editing sites, named A, B, E (C0), C and D [20], causing amino acid changes from isoleucine to valine (I/V) at positions 157 and 161, isoleucine to methionine (I/M) at position 157, asparagine to serine (N/S), asparagine to aspartate (N/D) and asparagine to glycine (N/G) at position 159. Editing does not occur simultaneously at all five sites, and the differential combination of editing results in at least seven 5-HT_2C_R protein isoforms in rats [20] and at least 12 in humans [21]. Coincidentally, all five editing sites reside closely in the second intracellular loop of the receptor [20], which is responsible for its coupling to the G-protein [22]. Radio-ligand binding and functional studies of the edited isoforms showed a reduction in G-protein coupling and a lower level of constitutive activity, suggesting that RNA editing may be a novel mechanism for regulating neuronal excitability by stabilizing receptor signaling and enhancing the signal to noise ratio at serotonergic synapses [21].

### Family of Adenosine Deaminases that Act on RNAs (ADARs)

Currently, three members of the ADAR family have been discovered in mammals, namely ADAR1, ADAR2 and ADAR3, according to the sequence of discovery. ADAR1 and ADAR2 were shown to be ubiquitously expressed, with enzymatic targets identified mainly in the nervous system while ADAR3 expression is restricted to brain and has yet no known targets [3,23]. ADARs work by recognizing partial or complete double-stranded RNA duplexes that are formed via base-pairing between the edited site and the editing-site complementary sequence (ECS), which is usually located in the downstream intron [24]. Although the mechanism of site-specific editing is incompletely understood, at least two studies have provided some insight into the roles of the multiple copies of double-stranded RNA binding motif (dsRBM). The dsRBM is a small motif of 75–85 amino acids, with the typical βαββαβ topology that forms a four stranded β-sheet packed against two α-helices [25]. ADAR1 contains three dsRBM, and ADAR2 and ADAR3 each contains two dsRBMs in the amino (N) -terminus.

**Figure 1:**
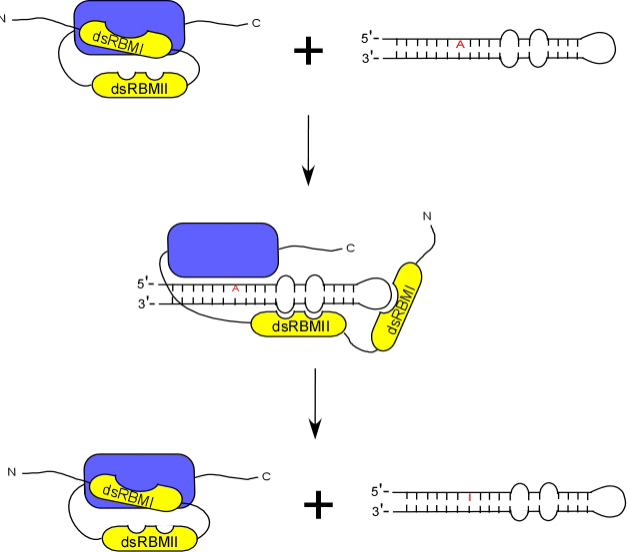
**Model of the conformational changes during ADAR2-mediated RNA editing**. The deaminase domain is represented by the blue box; double-strand RNA binding motif (dsRBM), by the yellow boxes. In the absence of RNA, ADAR2 protein is inactive because the N-terminal domain prevents the association of dsRBMII and catalytic domain with substrate. In the presence of RNA substrate with specific secondary elements and sufficient length, conformational change frees up the catalytic domain, and allows both dsRBM and catalytic domain to bind to RNA substrate, as well as activation of enzyme. After the edited site is converted from A (in red) to a G (in red) by ADAR2, the RNA substrate is released for translation. (Figure adapted from Macbeth et al [26].)

Full-length ADAR2 is unable to edit a 15-base pair RNA substrate harboring the R/G site of GluR-B, but this activity is restored when the N-terminal segment, including the dsRBM1 was deleted [26]. Furthermore, addition of the N-terminal segment *in trans *inhibits the recovery of editing activity, supporting the hypothesis that dsRBM1 inhibits the catalytic site of the enzyme. The study also implies that a minimum length requirement for the duplex RNA substrate exist, in order to engage both of the dsRBMs. Such a limitation would therefore restrict the editing specificity to RNA duplex structure of sufficient length. A second study reveals that the two dsRBMs of ADAR2 recognize different secondary structural elements of the RNA [27]. Via NMR-based model of the complex formed between ADAR2 protein and RNA encoding the R/G site, it is observed that while the dsRBM1 binds preferentially to the loop regions, the dsRBM2 recognizes two bulges in the stem region adjacent to the edited site (as shown in Figure 1). Therefore, recognition and binding of ADAR substrates by dsRBMs is structurally-dependent and length sensitive. In addition, multiple copies of dsRBMs and subtle amino acid changes among the different dsRBMs could allow preferential binding of ADAR family members to RNA substrates with different secondary structures.

Substrate specificity could also be defined by the deaminase domain of ADAR. Protein chimeras, in which the deaminase domains were exchanged between ADAR1 and ADAR2, retained the substrate specificity of the donor's deaminase domain [28]. Although the crystal structure of ADAR2's deaminase domain has been resolved, and the roles of zinc and inositol hexakisphosphate (IP6) as co-factors have been elucidated [29], the mechanism by which the catalytic site confers target specificity remains unknown.

ADAR proteins show exclusive nuclear localization and activity, with the exception of one splice variant of ADAR1, and edit the primary transcripts before splicing occurs [2]. While A-to-I editing in the coding region potentiates the generation of multiple RNA and protein isoforms from a single gene, editing in the untranslated regions may impact on other aspects of RNA functions such as splicing, mRNA stability and translation efficiency [30]. For example, editing in the intron may introduce alternative splice sites and could hence alter the reading frame.

### Regulation of ADAR activity by self-editing

Both the pre-mRNA and mRNA of rat ADAR2 are susceptible to A-to-I editing, mediated by ADAR2 itself [30]. The rADAR2 pre-mRNA self-editing occurs in intron 1 and exon 2, which comprise the hotspot of this gene, at six different editing positions, namely -2, -1, +10, +14, +23, and +24 [30,31]. Editing at position -1 in intron 1 converts an adenosine-adenosine (AA) dinucleotide to adenosine-inosine (AI), which mimics the canonical AG dinucleotides normally found at the 3' splice junction [30]. The presence of AI thus acts as an alternative 3' splice acceptor site, resulting in the retention of 47 nucleotides (nt) at +28 position of exon 1. This generates a frameshift and a truncated protein sequence with no editing activity, if translation starts at the first initiator methionine residue. Species differences is observed in the inclusion of 47-nt isoform in the brain, with an expression level of approximately 80% in mouse brain, while only around 15% of the ADAR2 transcripts in human brain has it [32]. In both species, the expression of the self-edited 47-nt isoform is more abundant in the brain than in the liver [32].

Alternatively, a downstream initiator methionine at amino acid position 25 in rat ADAR2 could be used to generate the functional protein for the self-edited isoforms [30]. However, in human ADAR2 proteins, the next methionine occurs only at position 84, which lies in the dsRBM1. Initiation of translation at this site may then compromise the RNA binding capacity of the expressed protein, and furthermore, may be inefficient and reduce protein expression. Transgenic mice lacking ADAR2 self-editing express significantly higher levels of ADAR2, accompanied by increase in the editing levels of various ADAR2 substrates [33]. This implies that editing of its own pre-mRNA may thus serve as a negative feedback mechanism by which ADAR2 regulates its own expression activity.

### Regulation of ADAR activity by alternative splicing

**Figure 2:**
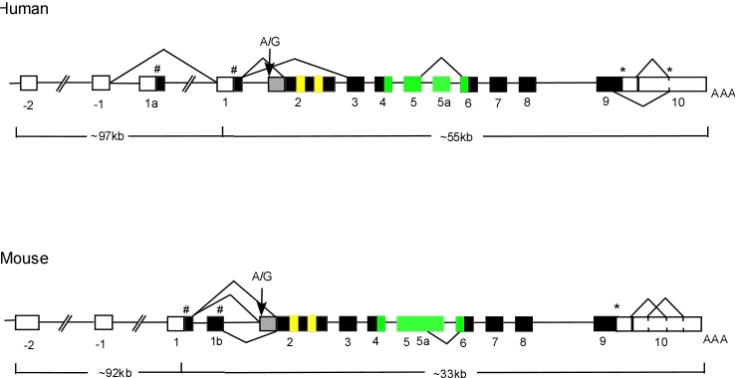
**ADAR2 genomic structures for both human and mice**. Exons are represented by boxes; intron, by lines. Filled boxes are coding and open boxes are non-coding. # and * indicate the positions of potential initiator methionines and stop codons respectively. The grey box before exon 2 indicates the 47 nucleotide cassette, and A/G denotes the site of editing that creates the AG splice site. Alternatively spliced exons are indicated. The yellow box indicates the position of dsRBM, double stranded RNA binding domain while the green box indicates the position of catalytic domain. (Figure adapted from Slavov and Gardiner [32].)

The human ADAR2 gene consists of 14 exons, with exons -2 and -1 located in the 5'-untranslated region (UTR) and exons 9 and 10 in the 3'-UTR [32]. In addition to retention of 47-nt via self-editing, multiple variations are also observed at the N-terminal via splicing, as indicated in Figure 2. In humans, the alternative inclusion of exon 1a adds 28 amino acids to the front of the commonly recognized initiator methionine residue. In comparison, the retention of exon 1b in mouse results in frame-shifting and early truncation of the protein [32]. Although it is proposed that alternative use of methionine in exon 1b may still result in the production of a functional protein, it is yet to be determined if the transcripts containing exon 1a or exon 1b could be expressed in vivo.

Maas and Gommans recently discovered a novel exon (exon 0) of mammalian ADAR2 located 18 kb upstream of exon 1, which extends the open reading frame by 49 amino acids [34]. Interestingly, the 49 amino acid extension harbors a sequence motif that is closely related to the R-domain of ADAR3, where it has been shown to function as a basic single-stranded RNA binding domain. Quantitative expression analysis shows that expression of the novel splice variant is tissue-specific, with the highest level in the cerebellum. In addition, the strong sequence conservation of the R-domain between human and rodent ADAR2 genes suggests a conserved function for this splice isoform.

Alternative splicing of exon 2 in the mRNA transcript of human ADAR2 could result in frame-shifting and an early stop codon in exon 3 [35]. In addition, the inclusion of an in-frame *Alu *sequence encoded by exon 5a in human ADAR2 reduces its catalytic activity by 50% [36]. In comparison, use of an alternative 3' splice acceptor site in intron 5 of mouse ADAR2 results in the inclusion of 30-nt *Alu *sequence and relatively higher catalytic activity [30].

Furthermore, use of the alternative splice site in exon 9 leads to a deletion of 29 amino acids from the carboxyl-terminal of human ADAR2 protein, resulting in a premature stop in exon 10. Such splicing event results in ADAR2 isoforms that have little editing activity on GluR-B mRNA [33]. Splicing in the 3' UTR of both human and mouse ADAR2 further contributes to the repertoire of ADAR2 proteins. Differential protein expression of C-terminal splice isoforms, detected by western blot, implies that such subtle splicing events could also play a role in regulating the translation efficiency of ADAR2 [35].

### Cross-talk among ADAR1, ADAR2 and ADAR3

ADAR1 and ADAR2 have been shown to function as homodimer by both *in vivo *and *in vitro *experiments while the endogenous dimer formation of ADAR3 is also observed in mouse brain [14]. However, there is still controversy regarding interaction between the different ADARs to form heterodimers [14]. Overexpression of ADAR1 is shown to inhibit the editing activity of ADAR2 in pediatric astrocytoma, and ADAR1 could co-immunoprecipitate with ADAR2, suggesting a possible mechanism of inhibition via sequestering ADAR2 by ADAR1 in a heterodimer formation [37].

Formation of heterodimers between ADAR splice isoforms is shown to influence the overall editing efficacy. A reduction in RNA editing efficacy is observed in transgenic *Drosophila *expressing both wild type and inactive ADAR isoforms, which retains the binding to dsRNA substrates [38]. Although the structural details of interaction between two ADAR monomers are still unknown, the crystal structure of structurally similar cytidine deaminase APOBEC-1 homodimer has indicated that the active site in each monomer is completed only with contribution from the other partner [36]. Therefore, it is possible that a heterodimer of ADAR1 and ADAR2 may result in a non-functional enzyme that could neither edit ADAR1 nor ADAR2 substrates. In addition, the combination of splice variants of ADAR2 with low or no editing activities could also exert dominant negative effect via the formation of heterodimer with the wild type enzyme.

Furthermore, ADAR3 is observed to inhibit the activities of ADAR1 and ADAR2 *in vitro*, suggesting a possible role of ADAR3 in regulating the editing activity mediated by ADAR1 and ADAR2 [39].

### Subcellular localization of ADAR1 and ADAR2

Subcellular localization of ADAR1 and ADAR2 proteins offers another intriguing aspect on the regulation of A-to-I editing efficacy. ADAR1 proteins, which shuttle between the nucleus and cytoplasm, contain a nuclear localization signal (NLS) located in the dsRBM3 [40] and a putative nuclear export signal (NES) in the N-terminal [41]. On the other hand, the activity of ADAR2 is restricted to the nucleus due to the single NLS in its N-terminal domain [42].

It is demonstrated by Desterro and co-workers that both ADAR1 and ADAR2 could be transiently sequestered into the nucleolus, a subnuclear region where transcription of ribosomal RNA (rRNA) occurs [41]. Local accumulation of small nucleolar RNAs (snoRNAs) in the nucleolus may be responsible for the tethering of ADAR1 and ADAR2, due to interaction between these dsRNA structures with the dsRBD of ADAR. Enhanced translocation of ADAR2 to the nucleoplasm occurs upon the inhibition of rRNA synthesis or the presence of editing substrates in the nucleoplasm, which correlates with increased editing efficacy [42]. Such a model would therefore suggest that the editing substrates and snoRNAs compete for binding to the ADAR proteins. Hence, with accumulation of specific editing substrates in the nucleoplasm, the balance shifts and ADAR proteins are recruited to the nucleoplasm for A-to-I editing.

### Developmental regulation of RNA editing

Editing of mRNA transcripts by ADAR enzymes is critical for normal life and development, as observed from the knockout mice that are deficient in either ADAR1 or ADAR2. ADAR2^-/- ^knockout mice develop normally, but are prone to early onset of epilepsy and die within three weeks of birth. Underediting of the Q/R site in the GluR-B transcripts appears to be the underlying reason for the epileptic seizures, which could be rescued by introducing an edited version of GluR-B gene into the ADAR2^-/- ^mice [43]. The deletion of ADAR1 in mice is embryonically lethal, and mice die between E11.5 and 12.5, as the mice develop a severe liver defect that involves cells of both the hematopoietic and hepatic lineages. Thus, ADAR1 appears to have a critical function in non-nervous tissues, which is likely to affect transcript editing [44].

Developmental analysis of known RNA editing substrates shows that editing of certain sites is regulated during the development of the brain. These would include mRNA transcripts of the AMPA and kainate members of the glutamate receptor family – GluR-B, GluR-5 & GluR-6 [33], the serotonin 5-HT_2C _receptor [45], the α3 subunit of GABA_A _receptor [16,17], and ADAR2 itself [30]. Stimulation of calcium-permeable AMPA receptors steers differentiation of neural progenitor cells (NPCs) preferentially to neuronal cells, and promotes dendritic arborization. Overexpression of ADAR2 in NPCs results in Q/R editing and expression of calcium-impermeable AMPA receptors, which prevents AMPA-mediated differentiation, suggesting a physiological role for the Q/R editing process and the importance of unedited AMPA receptor subunits in neurogenesis [46]. While transition to edited AMPA correlates with the overexpression of ADAR2, the exact mechanism and/or factors involved in regulation of RNA editing remain to be elucidated. Two studies tried to establish a correlation between ADAR mRNA expression level and editing frequency of 5-HT_2C_R and GluR-B transcripts [45,47], but concluded that changes in expression level only is an insufficient model.

In the rat brain, both developmental and regional regulation of ADAR2 mRNA expression and splicing are observed [47]. Two splice variants of ADAR2, differing by the addition of a 30-nt *Alu *cassette, colocalize throughout the brain. While expression in the thalamic nuclei is the highest, the *Alu*-containing splice variant is more abundant overall. Interestingly, the expression of ADAR2 mRNA does not correspond temporally or spatially with its physiological substrates, GluR-B and 5-HT_2C_R. A separate study by Hang *et al *finds that while expression level of ADAR2 mRNA markedly increases, that of ADAR1 mRNA remains constant during development [45]. During the embryonic period, ADAR2 pre-mRNA is unedited, but the number of edited sites and editing frequency increase after birth, suggesting that expression of edited ADAR2 may be significant to normal brain development. In addition, expression of ADAR1 and ADAR2 does not correspond with editing frequencies of specific target sites in the 5-HT_2C_R mRNA. ADAR1 edits the A- and B-sites in 5-HT_2C_R mRNA [48], while ADAR2 mediates editing of sites C and D [49]. Although the editing efficacies markedly increase during development, ADAR1 mRNA expression remains constant, suggesting that other factors may cooperate with ADAR1 such as RNA helicase [49,50].

Three models are proposed to help explain the mechanism behind the developmental increase in RNA editing [33]. First, ADAR1 and ADAR2 could be regulated at the post-transcriptional level such as protein modification or compartmentalization, which would enhance enzyme activity or its access to substrates. Second, the editing machinery may need a cofactor, which could synergistically enhance the actions of ADAR enzymes. Some potential candidates include the zinc ion, which coordinates and allows binding of ADAR2 to its pre-mRNA substrates, or the IP6, an inositol polyphosphate that binds and fills an extremely basic cavity in ADAR2 [29]. Third, access of ADAR enzymes to the editing sites may be controlled negatively by a competitive inhibitor. The 5-HT_2C_R mRNA has significant sequence complementarity with a small nucleolar RNA, MBII-52, which specifically inhibits nucleolar editing [51], and the relative expression of MBII-52 may shift the balance of editing by trafficking or sequestering transcripts to specific compartments [52].

Hence, although the mRNA level of ADAR1 and ADAR2 may play critical physiological roles in the brain and peripheral tissues, changes in the expression of these enzymes alone could not account for the developmental regulation of RNA editing observed in the brain.

### Activity-dependent RNA editing

RNA editing can be regulated by changes in metabolic activity, such as glucose concentration or serotonin level. Gan *et al *[53] reported an upregulation of ADAR2 transcripts and an increase in ADAR2-mediated editing of the GluR-B subunits in pancreatic β-islet cells exposed to physiological concentration of glucose, and the reverse occurred when the glucose concentration was decreased. Transient forebrain ischemia is caused by disruption of blood flow to the brain, causing a highly selective pattern of neuronal loss in the nervous system due to entry of toxic zinc and calcium ions into vulnerable neurons. In the setting of ischemia, heightened glycolysis could perturb glucose concentrations, leading to altered ADAR2 activity and editing. It is shown that forebrain ischemia in adult rats selectively reduces ADAR2 enzyme expression, and hence, disrupts editing of the Q/R site in GluR-B in vulnerable neurons [54]. Editing of this site determines the vulnerability of neurons in the rat hippocampus to forebrain ischemia. Expression of exogenous ADAR2 gene or a constitutively active CREB, which induces expression of endogenous ADAR2, results in recovery of Q/R editing in GluR-B and protection of vulnerable neurons in the rat hippocampus from this injury. In addition, ADAR2 gene silencing leads to degeneration of ischemia-insensitive neurons, and direct introduction of mutated gene, GluR-B^Q607R ^prevents neuronal degeneration.

As mentioned above, the 5-HT_2C_R pre-mRNA is edited at five closely-spaced adenosine residues located within the second intracellular domain of the receptor. The efficacy of editing at these sites is dependent on the serotonin level. Significantly decreased levels of editing at the C- and E- sites of 5-HT_2C_R mRNA are observed in serotonin-depleted mice, leading to the expression of receptors with higher sensitivity to serotonin [55]. However, treatment with a 5-HT_2A/2C _agonist increases the editing frequency at the E-site and the expression of receptor that activates G-proteins least efficiently. Hence, changes in the editing status of 5-HT_2C_R pre-mRNA could alter synaptic receptor activation and signaling bidirectionally depending on the receptor isoforms present.

Through the understanding of regulation of RNA editing in the physiological conditions, we could extend this knowledge into the study of pathophysiological conditions, and new treatment strategies that specifically target injured areas in the nervous system.

### Consequences of RNA editing dysregulation

Dysregulation of RNA editing has been linked to many human diseases and recent studies have shown its importance in cancer [56]. Patients with glioblastoma multiforme (GBMs) have a reduction in both ADAR2 activity and editing of the GluR-B mRNA at the Q/R site [57]. Neoplasm of glial cells represents the most common tumors of the CNS with GBMs being the most malignant [56]. Ishiuchi *et al *demonstrated that the Ca^2+^-permeable non-edited GluR-B^Q ^promotes migration and proliferation of the gliobastomas, thus driving invasion and abnormal growth [58]. Expression of the edited GluR-B^R^, which is less permeable to calcium ion, prevents cell migration and induces cell death. Therefore, the Q/R site of the GluR-B is necessary for regulating migration of glioblastoma cells.

In a separate study, a correlation between reduction of ADAR2 editing activity and grade of astrocytomas is observed in children [37]. The authors also found that restoration of the ADAR2 editing activity in astrocytoma cell lines inhibit cell migration and proliferation. Using both bioinformatic approach and direct sequencing, considerable global hypoediting of *Alu *repetitive elements is observed in various tumors of the brain, prostate, lung, kidney, and testis [59]. Reduction of ADAR3 is found to correlate with the malignancy of GBMs. In addition, the mRNA levels of all 3 members of the ADAR family are reduced in brain tumors, most significantly in ADAR3. Overexpression of ADAR1 and ADAR2 in GBM cell line results in a decreased proliferation rate, suggesting that reduced A-to-I editing in brain tumors is involved in the pathogenesis of cancer [59].

Dysregulation of epigenetic mechanisms is recently shown to play a central role in pathogenesis of cancer. Interestingly, A-to-I RNA editing may serve as an additional epigenetic mechanism relevant to cancer development and progression. In the mammalian nervous system, ion channels are common targets of ADARs-mediated editing. Ion channels play an important role in controlling cell cycle and proliferation and alteration of their activities may facilitate cancer progression [60]. However, as neoplastic cells are genetically unstable and exhibit numerous altered biological pathways, it is debatable as to whether reduction in editing of ADARs substrates is the cause or just a consequence of tumor progression.

Epilepsy is a neurological disorder characterized by recurrent, unprovoked seizures, due to neuronal hyperexcitability. The epileptic phenotypes observed in GluR-B editing-deficient mice are most profound and the severity of seizure activity correlates directly with the degree of impairment in editing of the Q/R position [61]. Although the molecular mechanism for epileptogenesis in these transgenic mice has not been elucidated, pre-editing of the GluR-B mRNA through genomic mutation rescues the seizure phenotype, suggesting that it is the changes in the AMPA channel properties are the cause. A conditional mouse mutant where GluR-B editing is inactivated postnatally in selected forebrain regions supports the notion that unedited GluR-B directly causes neuron hyperexcitability, as editing deficiency in adult mice induces seizures with similarity to human temporal lobe epilepsy [62]. In contrast, elimination of GluR-B, which also results in Ca^2+^-permeable AMPA receptors, does not elicit an epileptic phenotype in mice. Underediting of the Q/R site in GluR-B is critical because it not only determines AMPA receptors' Ca^2+ ^permeability, but also regulates gating kinetics, channel conductance, and channel assembly and trafficking. Epilepsy associated with GBM might also be linked to a decrease in GluR-B Q/R site editing [57].

Amyotrophic lateral sclerosis (ALS) is a progressive neurodegenerative disease of the motor neurons, resulting in progressive symptoms of muscle weakness, muscle atrophy, spasticity and eventually paralysis. Glutamate excitotoxicity appears to contribute to the pathology of sporadic ALS, whereby increase in glutamate levels suggest that prolonged activation of glutamate-gated ion channels might lead to excessive Ca^2+ ^influx and neuronal death. The contribution of excessive Ca^2+ ^influx through glutamate receptors to the death of motor neurons suggest that RNA editing might play a role in ALS. Editing at the Q/R position in GluR-B of spinal motor neurons from ALS patients were severely decreased, ranging from 62–100%, whereas all normal control cells showed 100% editing [63]. However, this deficiency in editing was not detected in motor neurons of rats transgenic for mutant human SOD1 (an animal model for familial ALS) or in patients with non-ALS motor neuron diseases, indicating that abnormal editing may be a contributory cause of neuronal death specifically in sporadic ALS. Again, it is not known when during the disease progression this impairment in editing arises or if it might be a causative event during the onset of ALS. Furthermore, this deficiency is only observed in spinal motor neurons, whereas upper motor neurons, which also degenerate in ALS, showed unaltered editing levels of GluR-B. This means that a defect in the Q/R site editing cannot be the sole mechanism responsible for the demise of the motor neurons. A recent genetic mouse model, whereby GluR-B subunits express the residue asparagine (N) at the Q/R position, results in channels with modestly increased Ca^2+ ^permeability and late-onset motor neuron disease resembling the pathological changes observed in sporadic ALS [64]. Substantial increase in Ca^2+ ^permeability of the AMPA receptors in principal neurons leads to early lethality precluding the observation of late-onset degenerative diseases. In addition, the GluR-B^N ^mutation accelerates disease progression and decrease survival in the background of SOD1^G93A ^mutation, which has been linked to cases of familial ALS.

### Clinical relevance of RNA editing: 5-HT_2C _receptor

The involvement of serotonin in psychiatric disorders such as schizophrenia, depression and anxiety has been based largely on pharmacological studies [65,66], while its action mechanism remains to be elucidated. Several studies suggest that editing of 5-HT_2C_R mRNA is involved in the pathophysiology of psychotic disorders, and may also regulate efficacy of hallucinogenic and antipsychotic drugs on the serotonin 5-HT_2C _receptors [21,55,67]. A marked reduction in sensitivity to LSD and atypical antipsychotic drugs is observed in the fully edited human 5-HT_2C_R (VGV) isoform, which suggests a role for edited 5-HT_2C_R in the etiology and pharmacotherapy of schizophrenia [21]. Cohort studies of depressed suicide victims and schizophrenic patients observe altered editing of 5-HT_2C_R pre-mRNA in the prefrontal cortices, but caution must be taken when interpreting these results. For instance, no alterations of editing patterns is observed in three independent studies conducted regarding schizophrenia [21,67,68], while only the report by Sodhi *et al *[69] claims to observe significant decrease in editing of the B-site of 5-HT_2C_R mRNA. This discrepancy could be due to limitations of such studies, namely the small sample size, methodological differences, and medication history of subjects. Interestingly, two separate studies by Niswender *et al*, and Iwamoto and Kato report increased RNA editing efficacy at the A-site of 5-HT_2C_R mRNA in depressed patients. Furthermore, Gurevich *et al *observe a significant increased at the E-site as well [55], which it also reported in a rat model of depression, and could be reverted through treatment with serotonin-selective reuptake blocker fluoxetine [67].

Among inbred strains of mice, the pattern of 5-HT_2C_R editing is found to be significantly different [70]. In C57BL/6 and 129SV mice, more than 80% of forebrain neocortical 5-HT_2C_R mRNAs are edited in at least one site while in the BALB/c strain, about 80% of the serotonin 2C receptors are not edited at all. Furthermore, the strains also differed in alteration of the editing pattern against acute stress or chronic treatment of fluoxetine. These observations imply that editing responsiveness to stress and medication is modulated by genetic background, as well as behavioral state. Thus, development of animal models may provide more robust insights into relationships between editing of serotonin 2C receptors and mental disorders as compared to association studies using postmortem brains.

A-to-I editing of 5-HT_2C_R mRNA mediated by ADARs may be involved in mental disorders, and the increased understanding of the editing mechanism and target spectrum can explain some of the phenotypic features that result from RNA editing deficiency or hyperactivity. Owing to the functional implications of RNA editing, the ADAR enzymes may be targets for the pharmacological treatment of diseases of the central nervous system, such as depression, epilepsy, and schizophrenia.

### Summary and outlook

RNA editing was once considered a rare peculiarity, and most studies in the mammalian brain have historically focused on just two editing targets, glutamate-gated ion channels and serotonin 2C receptors. However, a growing list of other brain editing targets has begun to overturn this initially limited view. RNA editing generates a diverse repertoire of proteins from a small fixed pool of genes that is able to fine tune and response dynamically to rapid changes in the nervous system. Dysregulation of RNA editing results in irregular responses in the nervous system, some of which could even prove fatal [43]. Current understanding of the mechanisms underlying the regulation of RNA editing is still poorly understood and any correlations are still hypothetical. This review attempts to provide an insight to existing models of explanation – regulation of ADAR expression and activity by self-editing, alternative splicing and cross-talk among ADAR family members; developmental and activity-dependent regulation of A-to-I editing through environmental cues. Besides identifying new targets of A-to-I editing through bioinformatics, focus should be redirected to methodically examining the regulation of editing, as it seems to contribute significantly to both the physiological and pathophysiological conditions observed in the mammalian nervous system.

## Competing interests

The authors declare that they have no competing interests.

## Authors' contributions

All authors participated in developing the ideas, the writing, discussion and integration of the information. All authors read and approved the final manuscript.

## Supplementary Material

Additional file 1 **ADAR substrates with editing sites in coding sequence**. The table summarizes currently known ADAR substrates with the editing sites named according to the amino acid change in the coding sequence, and the functional changes on the channel and receptor proteins. Click here for file
