# Supplementary material for: Dynamic regulation of RNA editing of ion channels and receptors in the mammalian nervous system
Source: Mol Brain. 2009 May 29;2:13. doi: 10.1186/1756-6606-2-13 (PMC2694175; doi:10.1186/1756-6606-2-13)
Supplement: Additional file 1 — ADAR substrates with editing sites in coding sequence. The table summarizes currently known ADAR substrates with the editing sites named according to the amino acid change in the coding sequence, and the functional changes on the channel and receptor proteins. [file 1756-6606-2-13-S1.doc]

Table 1: ADAR substrates with editing sites in coding sequence.

| **RNA** | **Base-pairing** | **Codon changes^a^** | **Functional changes** | **Reference** |
| --- | --- | --- | --- | --- |
| **Glutamate receptor** |  |  | **Editing of Q/R site lowers Ca^2+^ permeability, & receptor tends to be retained at ER as monomer Non-edited GluR-B^Q/Q^ causes epileptic seizures and death within 3 weeks of birth Editing of R/G site enhances recovery from desensitization Editing of Q/R site in kainate receptors potentiates inhibition of receptors by membrane fatty acids Editing of all 3 sites in gluR-6 increases higher Ca^2+^ permeability** |  |
| **gluR-B (AMPA)** | **Exon/intron** | **Q/R^607^, R/G^764^** |  | **[4, 8]** |
| **gluR-C (AMPA)** | **Exon/intron** | **R/G^769^** |  | **[8]** |
| **gluR-D (AMPA)** | **Exon/intron** | **R/G^765^** |  | **[8]** |
| **gluR-5 (kainate)** | **Exon/intron** | **Q/R^636^** |  | **[4]** |
| **gluR-6 (kainate)** | **Exon/intron** | **Q/R^621^, I/V^567^, Y/C^571^** |  | **[4, 7]** |
|  |  |  |  |  |
| **K_v_1.1 channel** | **Exon/exon** | **I/V^400^** | **Rapid recovery from inactivation, shortening duration of and increasing frequency of action potential** | **[13, 15]** |
| **GABA_A_-α3 receptor** | **Exon/intron** | **I/M^342^** | **Smaller peak current amplitudes, slower activation, and faster deactivation compared to non edited receptors** | **[16, 17]** |
| **Serotonin receptor** | **Exon/intron** | **I/V^157 & 161^, I/M^157^, N/D^159^, N/S^159^, N/G^159^** | **Lower coupling efficacy to G-protein Lower tendency to isomerizes, hence lower level of constitutive activity** | **[20, 21]** |
| ***^a^ Editing sites are named according to the amino acid change they produced and amino acid position (unedited/edited ^[amino acid position]^)*** | | | | |
